# Analysis of *PALB2* Gene in *BRCA1*/*BRCA2* Negative Spanish Hereditary Breast/Ovarian Cancer Families with Pancreatic Cancer Cases

**DOI:** 10.1371/journal.pone.0067538

**Published:** 2013-07-23

**Authors:** Ana Blanco, Miguel de la Hoya, Ana Osorio, Orland Diez, María Dolores Miramar, Mar Infante, Cristina Martinez-Bouzas, Asunción Torres, Adriana Lasa, Gemma Llort, Joan Brunet, Begoña Graña, Pedro Perez Segura, María José Garcia, Sara Gutiérrez-Enríquez, Ángel Carracedo, María-Isabel Tejada, Eladio A. Velasco, María-Teresa Calvo, Judith Balmaña, Javier Benitez, Trinidad Caldés, Ana Vega

**Affiliations:** 1 Fundación Pública Galega de Medicina Xenómica-Servicio Galego de Saúde, Grupo de Medicina Xenómica-Universidade de Santiago de Compostela, Spanish Network on Rare Diseases (CIBERER), Instituto de Investigaciones Sanitarias de Santiago, Santiago de Compostela, A Coruña, Spain; 2 Laboratorio de Oncología Molecular, Hospital Clínico San Carlos, Madrid, Spain; 3 Human Genetics Group, Human Cancer Genetics Programme, Spanish National Cancer Research Centre, Spain and Spanish Network on Rare Diseases (CIBERER), Madrid, Spain; 4 Oncogenetics Laboratory, University Hospital Vall d'Hebron, Vall d'Hebron Institute of Oncology (VHIO), Vall d'Hebron Institut de Recerca (VHIR), Universitat Autònoma de Barcelona, Barcelona, Spain; 5 Sección de Genética, Servicio de Bioquímica Clínica, Hospital Universitario Miguel Servet, Zaragoza, Spain; 6 Cancer Genetics, Instituto de Biología y Genética Molecular de la Universidad de Valladolid (UVA) y del Consejo Superior de Investigaciones Científicas (CSIC), Valladolid, Spain; 7 Laboratorio de Genética Molecular-Servicio de Genética, Hospital Univeristario Cruces, Bilbao, Spain; 8 Unitat de Consell Genètic. Hospital Universitari Sant Joan, Institut d'Investigació Sanitària Pere Virgili (IISPV), Reus, Spain; 9 Servei de Genètica, Hospital de la Santa Creu i Sant Pau, Barcelona, Spain; 10 Unidad de consejo genético, Institut Oncologic del Valles, Sabadell, Terrassa, Spain; 11 Hereditary Cancer Program, Catalan Institute of Oncology, Girona Biomedical Research Institute, Girona, Spain; 12 Oncoloxía Médica, Unidade de alto risco en cancro-Consello Xenético, Hospital Arquitecto Marcide, Área Sanitaria de Ferrol, A Coruña, Spain; 13 Medical Oncology Department, Hospital Clínico San Carlos, Madrid, Spain; 14 Medical Oncology Department, University Hospital Vall d'Hebron, Barcelona, Spain; 15 Human Genetics Group and Genotyping Unit, Human Cancer Genetics Programme, Spanish National Cancer Research Centre, Spain and Spanish Network on Rare Diseases (CIBERER), Madrid, Spain; Ohio State University Medical Center, United States of America

## Abstract

**Background:**

The *PALB2* gene, also known as *FANCN*, forms a bond and co-localizes with BRCA2 in DNA repair. Germline mutations in *PALB2* have been identified in approximately 1% of familial breast cancer and 3–4% of familial pancreatic cancer. The goal of this study was to determine the prevalence of *PALB2* mutations in a population of *BRCA1/BRCA2* negative breast cancer patients selected from either a personal or family history of pancreatic cancer.

**Methods:**

132 non-*BRCA1/BRCA2* breast/ovarian cancer families with at least one pancreatic cancer case were included in the study. *PALB2* mutational analysis was performed by direct sequencing of all coding exons and intron/exon boundaries, as well as multiplex ligation-dependent probe amplification.

**Results:**

Two *PALB2* truncating mutations, the c.1653T>A (p.Tyr551Stop) previously reported, and c.3362del (p.Gly1121ValfsX3) which is a novel frameshift mutation, were identified. Moreover, several *PALB2* variants were detected; some of them were predicted as pathological by bioinformatic analysis. Considering truncating mutations, the prevalence rate of our population of *BRCA1/2*-negative breast cancer patients with pancreatic cancer is 1.5%.

**Conclusions:**

The prevalence rate of *PALB2* mutations in non-*BRCA1/BRCA2* breast/ovarian cancer families, selected from either a personal or family pancreatic cancer history, is similar to that previously described for unselected breast/ovarian cancer families. Future research directed towards identifying other gene(s) involved in the development of breast/pancreatic cancer families is required.

## Introduction

Hereditary breast cancer accounts for approximately 5–10% of all breast cancer cases. Mutations in the two main susceptibility genes *BRCA1* and *BRCA2*, together with mutations in a number of other high-penetrance genes such as *TP53* and *PTEN*, account for 20% of familial breast cancer cases [1]–[3]. For the remaining 80%, the genetic factors are largely unknown and they are likely to involve mutations in moderate and low penetrance susceptibility genes, plausibly acting together with some environmental or other hereditary factors. Apart from breast and ovarian cancer, *BRCA1* and *BRCA2* carriers might be at higher risk for additional malignancies such as prostate, colorectal, familial melanoma and pancreatic cancers.

Pancreatic cancers are the fourth most common cause of cancer-related deaths in the Western world. Approximately 5% to 10% of individuals with pancreatic cancer report a history of pancreatic cancer in a close family member. In addition to this, several known genetic syndromes have been shown to be associated with an increased risk of pancreatic cancer. Thus, germline mutations in the *BRCA2*, *p16/CDKN2A*, *STK11*, and *PRSS1*, that are responsible for familial breast cancer, Familial atypical multiple melanoma, Peutz-Jeghers and Familial pancreatitis, respectively, have been clearly associated with an increase risk of pancreatic cancer [4]–[7]. Additionally, some studies have described pancreatic cancer developing among individuals with HNPCC [8], [9].

The *PALB2* (partner and localizer of BRCA2) gene was identified by searching for novel components of endogenous BRCA2-containing complexes [10]. PALB2 supports BRCA2 stability and determines its localization in the nucleus after DNA damage [10]. Relocation of PALB2 and BRCA2 to damaged chromatin is regulated by BRCA1. These three proteins form a complex in which PALB2 acts as a bridge between BRCA1 and BRCA2 [11]. This complex is critical for the initiation of homologous recombination in the DNA-damage response [11], [12]. In cells depleted of PALB2 the DNA repair pathway dependent on the BRCA1/2 is disrupted [10], [11]. Immediately after *PALB2* was discovered, evidence showed that it was also a Fanconi anemia gene, known as *FANCN* [13], [14]. Biallelic inactivation mutations in *PALB2/FANCN* cause Fanconi anemia subtype N, characterized by a severe predisposition to pediatric malignancies such as Wilms tumor, medulloblastoma, AML and neuroblastoma [14]. Interestingly, the gene underlying the D1 subtype of Fanconi anemia, *FANCD1* [15], was found to be *BRCA2* and biallelic mutations in *BRCA2/FANCD1* originate a phenotype with high risk of childhood malignancies, very similar to that produced by *PALB2/FANCN* biallelic mutations. This supports the proposal that PALB2 is important for BRCA2 tumor suppression activity [16].

As in other Fanconi anemia genes, monoallelic mutations in *PALB2* have been associated with increased breast cancer risk [3]. Thus, *PALB2* monoallelic mutations have been identified in approximately 1% of hereditary breast cancer families globally, as summarized by Tischkowitz and Xia [16]. It has recently become clear that the *PALB2* gene should not only be considered as a susceptibility gene for breast cancer but also for pancreatic cancer. This pancreatic association was based on the identification of a *PALB2* mutation by exomic sequencing and the subsequent *PALB2* analysis in additional familial pancreatic cancer patients that revealed a prevalence of 3.1% [17]. A similar prevalence (3.7%) was found by Slater et al [18] in European patients with familial pancreatic cancer, whereby *PALB2* carriers also had a history of breast cancer.

Given these findings, we aimed to determine the prevalence of *PALB2* mutations in a Spanish population of *BRCA1*/*BRCA2*-negative breast/ovarian cancer families with either a personal or family history of pancreatic carcinoma.

## Materials and Methods

### Patients

Index cases from 132 *BRCA1/BRCA2* mutation-negative unrelated Spanish breast/ovarian cancer families with a personal history of both breast and pancreatic cancer, or a family history with pancreatic cancer cases, were screened for mutations within the entire coding sequence and splicing sites as well as large genomic rearrangements of *PALB2* gene. Patient and family characteristics are summarised in Table 1. Families were enrolled from 11 different Spanish centres (Table S1). Ethical committee approval and informed consent for all participants in the study were obtained.

**Table 1:** *BRCA1/BRCA2* mutation-negative Spanish high risk breast/ovarian cancer families with pancreatic cancer cases.

Type of case		N° of cases (n = 132)	Mean age at cancer diagnosis	Additional family history
Personal history of BC and PC		3	BC:43.6	PC in FDR:0
			PC:44	PC in SDR:2
				BC in FDR:0
				BC in SDR:1
Personal history of PC and familiar history of BC and PC		4	PC:65	PC in FDR:1
			PC in FDR: 50	PC in SDR:0
				BC in FDR:4 (1BiBC)
				BC in SDR:1
Personal history of OC and familiar history of PC		9 (1BiOC)	OC:43.6	PC in FDR:3
			PC in FDR: 71.5	PC in SDR:7
			PC in SDR: 64.7	BC in FDR:1
				BC in SDR:2 (1 BiBC)
				OC in FDR:2
				OC in SDR:2
Personal history of PrC and familiar history of BC and PC		2	PrC:54.5	PC in FDR:1
			PC in FDR: 61	PC in SDR:2
			PC in SDR: 77	BC in FDR:2
				BC in SDR:2 (1 BiBC)
				OC in SDR:1
Personal history of BC and familiar history of PC	BC iagnosed <50	92 (13BiBC (1+leukemia, 1+melanoma; 1+OC), 1 MBC, 1+CCR	BC:39.2	PC in FDR:25 (1+BC)
			PC in FDR:58.8	PC in SDR:50
			PC IN SDR: 63.8	BC in FDR:31 (2 BiBC)
				BC in SDR:26 (1 BiBC, 1 MBC)
				OC in FDR:1
				OC in SDR:4
	BC diagnosed >50	22 (4 BiBC, 1MBC, 1+OC, 1+endometrium)	BC:58.6	PC in FDR:9 (1+BC)
			PC in FDR:64.1	PC in SDR:12 (1+BC)
			PC in SDR:65.7	BC in FDR:15 (1 BiBC, 1 MBC, 1+PC)
				BC in SDR:15
				OC in FDR:1
				OC in SDR:1

BC: Breast cancer; PC: Pancreatic cancer; OC: Ovarian cancer; BiOC: Bilateral Ovarian cancer; PrC: Prostate cancer; MBC: Male Breast cancer; BiBC: Bilateral Breast Cancer; CCR: Colorectal cancer; FDR: First degree relative; SDR: Second degree relative.

All index cases had been previously screened for point mutations and large rearrangements in *BRCA1* and *BRCA2* genes. All were found to be negative.

### Mutation analysis of the *PALB2* gene

Mutational analysis of *PALB2* gene included the complete coding sequencing and flanking intron-exon boundaries along with the analysis of genomic rearrangements, as previously described by Blanco et al [19].

### Nomenclature and databases

Sequences used for *PALB2* nomenclature were obtained from the NCBI RefSeq database (NG_007406.1 for genomic, NM_024675.3 for mRNA and NP_078951.2 for protein) (http://www.ncbi.nlm.nih.gov). Standardized nomenclature was reported considering the A of the ATG initiation codon of the coding DNA Reference Sequence as nucleotide position +1.

### Prediction of pathogenicity of splicing and missense variants

Splicing predictions were performed with Splicing Sequences Finder (SSF) (http://www.umd.be/searchSpliceSite.html), MaxEntScan (http://genes.mit.edu/burgelab/maxent/Xmaxentscan_scoreseq.html), NNSplice (http://www.fruitfly.org/seq_tools/splice.html) and HumanSplicingFinder (HSF) (http://www.umd.be/HSF/) algorithms through the Alamut- Mutation Interpretation Software v.1.54 (http://www.interactivebiosoftware.com/alamut.html). Default thresholds were used for all the analyses.

Potential consequences of missense substitutions were obtained using the prediction software PolyPhen-2 (Polymorphism Phenotyping-2, see http://genetics.bwh.harvard.edu/pph2/), SIFT (Sorting Intolerant From Tolerant, see http://sift.jcvi.org/) and Align-GVGD (Grantham score difference, see http://agvgd.iarc.fr/) tools. Native alignments of each algorithm were used (see Text S1 for a brief description of the tools).

CEU-population data from 1000 Genomes Project database (http://www.1000genomes.org/) was used to obtain allelic frequency of the identified variants in our samples.

## Results

**Figure 1:**
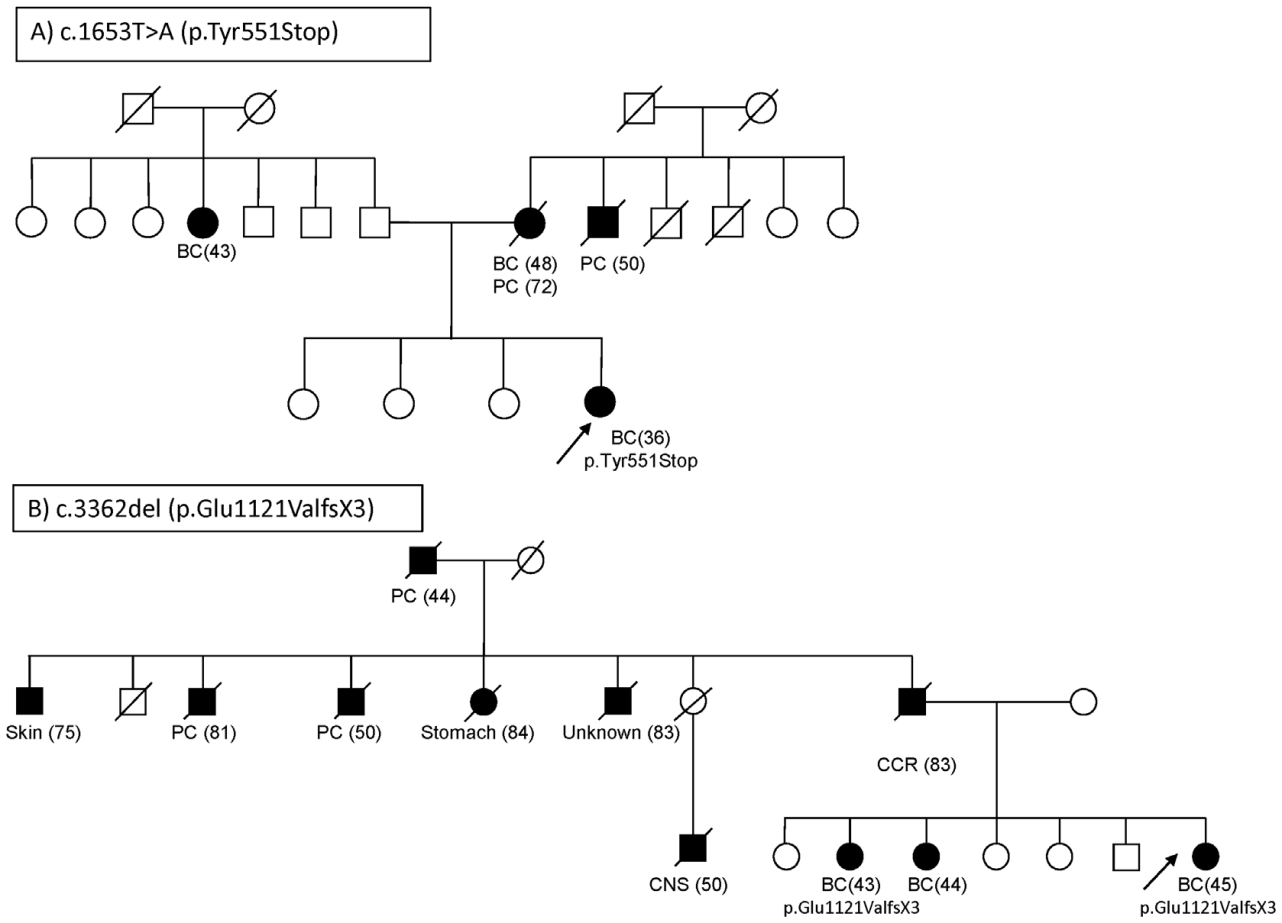
Pedigrees of the families carrying the mutations. a) c.1653T>A (p.Tyr551Stop); b) c.3362del (p.Glu1121ValfsX3). Cancer diagnoses are indicated in affected patients; in brackets the age at diagnosis or exitus. The *arrow* indicates the index case analyzed. *BC* breast cancer, *PC* Pancreatic Cancer, *CCR* Colorectal cancer, *CNS* central nervous system cancer.

We sequenced all exons and splicing boundaries of *PALB2* gene. We also carried out MLPA analysis in 132 index cases from *BRCA1/BRCA2*-negative families with breast and/or ovarian cancer with either a personal or familial history of pancreatic cancer. Two mutations were identified by sequencing analysis, the nonsense c.1653T>A (p.Tyr551Stop) located at exon 4 with the result of a premature stop codon, and the frameshift c.3362del (p.Gly1121ValfsX3) in exon 13, which is predicted to generate a translation-stop three codons downstream from the first affected amino acid. The *PALB2* truncating mutation c.1653T>A (p.Tyr551Stop) was identified in a woman diagnosed with an infiltrating ductal carcinoma (IDC), negative for estrogen receptor (ER), progesterone receptor (PR) and HER2 at the age of 36. Her mother had been diagnosed with breast cancer at 48 and with pancreatic cancer at 72 years of age. Her maternal uncle had been diagnosed with pancreatic cancer at 50 years of age (Figure 1A). Unfortunately, it was not possible to obtain samples from family members to confirm the mutation in the paternal branch of the proband. The frameshift mutation in exon 13, c.3362del produces a stop codon in position 1123 (p.Gly1121ValfsX3) that would cause the loss of the 63 amino acids from the N-terminal PALB2 region. Other truncated mutations in the last codons of the gene have already been described in breast/pancreatic cancer families [3], [20]. It has been shown that residues 836 to 1186 of the PALB2 protein are part of the WD40 repeats C-terminal domain which associates with the N-terminus of BRCA2 [12], [13]. The mutation c.3362del was identified in a woman diagnosed with breast cancer at the age of 45. Likewise, the same mutation was also detected in one of her sisters who was diagnosed with breast cancer at the age of 43. Furthermore, they had another sister who had been diagnosed with breast cancer; however, it was not possible to carry out the genetic analysis. The three of them were diagnosed with ER and PR positive infiltrating ductal carcinoma (IDC). Moreover, two paternal aunts and also the paternal grandfather were diagnosed with pancreatic cancer at the ages of 81, 50 and 44, respectively. We could not, unfortunately, confirm the origin of the mutation in the maternal branch. Other types of cancer present in the family were skin, stomach and CNS in paternal aunts (Figure 1B).

In addition to these two mutations, sequence analysis revealed another 21 different *PALB2* variants and polymorphims (Table 2), one in the 5′UTR region, 4 in introns and 16 in exons (12 missense and 4 silent coding variants). From the total number of variants identified, seven were novel (c.110G>A (p.Arg37His), c.212-180T>G, c.232G>A (p.Val78Ile), c.262C>T (p.Leu88Phe), c.1431T>C (p. = ), c.2587-59T>C, c.2837C>G (p.Ala946Gly)) and observed only once in our samples, whereas the other variants had been previously reported [3], [19], [21], [22].

**Table 2:** *PALB2* sequence variants identified in 132 Spanish breast/ovarian cancer families with pancreatic cancer cases.

				CASE POPULATION					CONTROL POPULATION	
				GENOTYPES			ALLELIC FREQUENCY (%)^b^		1000 GENOMES	
	NUCLEOTIDE CHANGE^a^	PROTEIN CHANGE	Name of SNP	AA	AB	BB	A (A%)	B (B%)	A%	B%
5′ upstream sequence	c.-47G>A	-	rs8053188	127	5	0	259(98.1)	5(1.9)	98	2
EXON 3	**c.110G>A**	**p.Arg37His**	**rs202194596**	131	1	0	263(99.6)	1(0.4)	100	0
INTRON 3	**c.212-180T>G**	**-**	**-**	131	1	0	263(99.6)	1(0.4)		
	c.212-58A>C	-	rs80291632	123	9	0	255 (96.6)	9(3.4)	96	4
EXON 4	**c.232G>A**	**p.Val78Ile**	**-**	131	1	0	263(99.6)	1(0.4)		
	**c.262C>T**	**p.Leu88Phe**	**-**	131	1	0	263(99.6)	1(0.4)		
	c.656A>G	p.Asp219Gly	rs45594034	131	1	0	263(99.6)	1(0.4)	100	0
	c.1010T>C	p.Leu337Ser	rs45494092	131	1	0	263(99.6)	1(0.4)	98	2
	c.1194G>A	p. =	rs61755173	130	2	0	262(99.2)	2(0.8)	100	0
	**c.1431T>C**	**p. = **	**-**	131	1	0	263(99.6)	1(0.4)		
	c.1572A>G	p. =	rs45472400	131	1	0	263(99.6)	1(0.4)	100	0
	c.1653T>A	p.Tyr551Stop	rs118203997	131	1	0	263(99.6)	1(0.4)		
	c.1676A>G	p.Gln559Arg	rs152451	105	24	3	234(88.6)	30(11.4)	90	10
EXON 5	c.2014G>C	p.Glu672Gln	rs45532440	122	10	0	254(96.2)	10(3.8)	96	4
INTRON 6	c.2586+58C>T	-	rs249954	90	37	5	217(82.2)	47(17.8)	79	21
	**c.2587-59T>C**	**-**	**-**	131	1	0	263(99.6)	1(0.4)		
EXON 7	c.2590C>T	p.Pro864Ser	rs45568339	129	3	0	261(98.9)	3(1.1)	100	0
EXON 8	c.2794G>A	p.Val932Met	rs45624036	131	1	0	263(99.6)	1(0.4)	99	1
	c.2816T>G	p.Leu939Trp	rs45478192	131	1	0	263(99.6)	1(0.4)	100	0
EXON 9	**c.2837C>G**	**p.Ala946Gly**	**-**	131	1	0	263(99.6)	1(0.4)		
	c.2993G>A	p.Gly998Gln	rs45551636	125	7	0	257(97.3)	7(2.7)	98	2
EXON 12	c.3300T>G	p. =	rs45516100	123	9	0	255 (96.6)	9(3.4)	96	4
EXON 13	**c.3362del**	**p.Gly1121ValfsX3**	**-**	131	1	0	263(99.6)	1(0.4)		

a In bold variants not previously reported.

b Allelic frequency is the percentage of n/N, where n is the number of minor alleles and N is the total number of alleles.

The results of bioinformatic predictions for intronic and missense variants are represented in Table 3. Four of the missense variants, c.1010T>C (p.Leu337Ser), c.2816T>G (p.Leu939Trp), c.2837C>G (p.Ala946Gly) and c.2993G>A (p.Gly998Gln), were predicted to likely affect PALB2 protein function by all the tested algorithms. Variants c.2816T>G (p.Leu939Trp) and c.2837C>G (p.Ala946Gly) are not present in CEU 1000 Genome Project samples, whereas c.1010T>C (p.Leu337Ser) and c.2993G>A (p.Gly998Gln), have a 2% frequency in CEU population (Table 2). For variant c.2816T>G (p.Leu939Trp), a similar frequency in controls and cases was observed [22]–[24]. For the missense variant c.110 G>A (p.Arg37His) two of the three prediction programs considered the variant as deleterious, whereas only one prediction tool considered variants c.262 C>T (p.Leu88Phe), c.2014 G>C (p.Glu672Gln), c.2590 C>T (p.Pro864Ser) and 2794 G>A (Val932Met) as deleterious (see Table 3). The bioinformatic splicing analyses showed consensus site score variations for variants c.110 G>A (p.Arg37His) (three programs), c.2837C>G (p.Ala946Gly) (two programs) and c.2993G>A (p.Gly998Gln) (one program). A destruction of a cryptic splice site was predicted for variants c.-47G>A and c.2794G>A (p.Val932Met) by three and two programs, respectively, as well as an increase in the score of a cryptic sites by all programs for the variant c.2794G>A (p.Val932Met) and by two programs for the variant c.2590C>T (p.Pro864Ser).

**Table 3:** Results of bioinformatic analysis for *PALB2* variants.

		PREDICTION AMINOACIDIC CHANGE			SPLICE SIGNAL DETECTION		
	PROTEIN CHANGE	POLYPHEN	SIFT	A-GVGD	Location, SS, Distance†	5′ or 3′ score modification (% variation)	Proximal Cryptic/De novo (% variation)
c.-47G>A	-	N/A	N/A	N/A	5′UTR, 5′, 47		c.-46 MaxEnt: 74.92→- (−100%)
							c.-46 SSF: 5.71→- (−100%)
							c.-46 HSF: 83.95→- (−100%)
c.110G>A&	p.Arg37His	Probably damaging	Affect protein function	Class C25	Exon 3, 3′, 2	MaxEnt: 10.06→9.64 (−4.3%)	
						NNSPLICE: 0.92→0.91 (−1.0%)	
						HSF: 82.10→81.93 (−0.2%)	
c.212-180T>G	-	N/A	N/A	N/A	Intron 3, 3′, 180		
c.212-58A>C	-	N/A	N/A	N/A	Intron 3, 3′, 58		
c.232G>A	p.Val78Ile	Benign	Tolerated	Class C25	Exon 4, 3′, 21		
c.262C>T	p.Leu88Phe	Possibly damaging	Tolerated	Class C15	Exon 4, 3′, 51		
c.656A>G	p.Asp219Gly	Benign	Tolerated	Class C65	Exon 4, 3′, 444		
c.1010T>C	p.Leu337Ser	Probably damaging	Affect protein function	Class C65	Exon 4, 5′, 674		
c.1194G>A	p. =	N/A	N/A	N/A	Exon 4,5′, 490		
c.1431T>C	p. =	N/A	N/A	N/A	Exon 4, 5′, 253		
c.1572A>G	p. =	N/A	N/A	N/A	Exon 4, 5′, 111		
c.1653T>A	p.Tyr551Stop	N/A	N/A	N/A	Exon 4, 5′, 31		
c.1676A>G	p.Gln559Arg	Benign	Tolerated	Class C35	Exon 4, 5′, 8		
c.2014G>C	p.Glu672Gln	Probably damaging	Tolerated	Class C25	Exon 4, 3′, 329		
c.2586+58C>T	-	N/A	N/A	N/A	Intron 6, 5′, 58		
c.2587-59T>C	-	N/A	N/A	N/A	Intron 6, 3′, 59		
c.2590C>T	p.Pro864Ser	Benign	Tolerated	Class C65	Exon 7, 3′, 4		c.2597 MaxEnt: 1.70→1.89 (+11.2%)
							c.2597 NNSPLICE: 0.53→0.65 (+22.3%)
							c.2597 HSF: 85.31→84.89 (−0.5%)
c.2794G>A	p.Val932Met	Probably damaging	Tolerated	Class C15	Exon 8, 5′, 46		c.2793 MaxEnt: 1.44→- (−100%)
							c.2793 HSF: 74.27→- (−100%)
							c.2795 SSF: −→72.85 (+100%)
							c.2795 MaxEnt: 1.30→2.60 (+100.7%)
							c.2795 HSF: 74.31→79.17 (+6.5%)
c.2816T>G	p.Leu939Trp	Probably damaging	Affect protein function	Class C55	Exon 8, 5′, 18		
c.2837C>G	p.Ala946Gly	Possibly damaging	Affect protein function	Class C55	Exon 9, 3′, 3	MaxEnt: 8.14→7.33 (−9.9%)	
						NNSPLICE: 0.54→- (−100%)	
c.2993G>A	p.Gly998Gln	Probably damaging	Affect protein function	Class C65	Exon 9, 5′, 4	NNSPLICE: 0.99→1.00 (+1.0%)	
c.3300T>G	p. =	N/A	N/A	N/A	Exon 12, 5′, 50		
c.3362del	p.Gly1121ValfsX3	N/A	N/A	N/A	Exon 13, 3′, 12		

The table reports 5′ or 3′ score modifications due to the detected variants in *PALB2* (for greater clarity, when the variants didn't change the score, the corresponding tool is not indicated). Proximal cryptic sites are indicated with the corresponding tool, when the variants led to *de novo* site it is also indicated. N/A = not applicable as the change is synonymous or intronic.

† Location indicates exon/intron, SS stands for splice site and distance to the nearest splice site is indicated in base pairs.

& cDNA analysis was performed. No additional products in the carrier sample compared to control samples has been identified (data not shown).

## Discussion

Mutations in *PALB2* gene were originally associated with an increased risk for breast cancer and later, with pancreatic cancer. We analyzed a large series of hereditary breast/pancreatic cancer families analysed for *PALB2* mutations. We identified two germline truncating mutations, the nonsense c.1653T>A (p.Tyr551Stop) located at exon 4 and the novel frameshift mutation c.3362del (p.Gly1121ValfsX3) in exon 13. These mutations were considered to be pathogenic, since they all create a stop codon that is predicted to cause a truncation of the *PALB2* protein. The nonsense mutation c.1653T>A (p.Tyr551Stop) had been previously reported in a Fanconi anemia patient as well as in a familial breast cancer case [13], [25]. Truncating mutations in *PALB2* are rare in individuals without cancer. In fact, they had not been identified in 1084 healthy individuals analysed [3]. In our series, c.1653T>A (p.Tyr551Stop) and c.3362del (p.Gly1121ValfsX3) were identified in index cases diagnosed with breast cancer under 50 years of age and at least one first degree relative diagnosed with pancreatic cancer. No ovarian cancer was present in these families. Considering these two *PALB2* variants as causal mutations, the prevalence of *PALB2* mutation in our *BRCA1/BRCA2* breast and pancreatic cancer series is 1.5% (2/132). Previous studies of breast/pancreatic cancer families have described prevalences from 0% (77 families analysed in Stadler et al [26], 45 in Adank et al [27], 29 in Guiorzo et al [28] and 28 in Harinck et al [29]), 2.1% (Hofstatter et al [24], 2 mutations in 94 families), to 4.8% (Peterlongo et al [20], 3 mutations in 62 families) reviewed in Table 4. Considering these studies with our data, the global prevalence of *PALB2* mutation in breast/pancreatic cancer families is 1.5% (7 mutations in 467 families).

**Table 4:** Overview of literature on role of *PALB2* mutations in FPC and in BC families affected by PC (BC-PC families).

STUDY	COUNTRY/ETHNICITY	PATIENTS	*PALB2* MUTATIONS	FAMILIAR HISTORY
Jones et al [17]	USA	96 FPC families	**c.172-175delTTGT**: 1(PC) **c.3116delA**: 1(PC+PrC) **c.3256C>T**: 1(PC+BC)	**c.172-175delTTGT**: family history of BC **c.3116delA**: family history of BC-PC **c.3256C>T**: family history of BC-PC
Tischkowitz et al [23]	Canada	254 PC (114 sporadic, 80 FPC, 21 FPC with breast/ovary cases; 39 sporadic PC with breast/ovary cases)	**6.7-kb germline deletion** (exons12–13): 1 (BC+PC)	**6.7-kb germline deletion** (exons12–13): family history of PC
Hofstatter et al [25]	USA	94 BC-PC families	**c.2962C>T**: 1BC(49) **c.3549C>CA**: 1BC(43)	**c.2962C>T**: family history of BC-PC **c.3549C>**CA: family history of BC-PC
Slater et al [18]	Europe	81 FPC families	**c.1240C>T**: 1(PC) **c.508-509delAG**: 1(PC) **c.3116delA**: 1(PC)	**c.1240C>T**: family history of BC-PC **c.508-509delAG**: family history of BC-PC **c.3116delA**: family history of BC-PC
Adank et al [28]	The Netherlands	45 BC-PC families (all index cases BC)		
Peterlongo et al [21]	Italy	62 BC-PC families	**c.72delG**: 1(BC) **c.1027C>T**: 1(BC) **c.3497delG**: 1(BC)	**c.72delG**: family history of BC-PC **c.1027C>T**: family history of BC-PC **c.3497delG**: family history of BC-PC
Stadler et al [27]	USA	77 BC-PC families		
Guiorzo et al [29]	Italy	29 BC-PC famililes		
Harinck et al [30]	The Netherlands	56 (28 FPC families as 28 BC-PC families)		
Current study	Spain	132 BC-PC families	**c.1653T>A**: 1(BC) **c.3362del**: 1(BC)	**c.1653T>A**: family history of BC-PC **c.3362delG**: family history of BC-PC

BC (breast cancer); PC (pancreatic cancer); FPC (familial pancreatic cancer); PrC (prostate cancer).

We recently, estimated a *PALB2* mutation prevalence of 0.75% for Spanish breast/ovarian cancer families with at least one male breast cancer case [19]. Although we identified twice as many carriers in families with pancreatic cancer cases than in families with male breast cancer cases, both prevalences are similar to the 1% reported for families with breast cancer unselected for other cancers [3], [30], [31]. Importantly, for most of the breast/pancreatic cancer series analysed, index cases were breast cancers. Similarly, in our study only 7 from the 132 index cases were pancreatic cancer. The selection of index cases could therefore be introducing a bias in the estimate of the prevalence of *PALB2* mutations in these families.

The selection of a gene to be included in a routine genetic test would be, in part, based on the risk it confers. However, the risk associated with deleterious mutations in genes like *PALB2* is not easily determined since deleterious alleles are extremely rare in the population, and the number of mutation carriers in published studies are small [32]. Mutations in *PALB2* gene were originally associated with a moderate (2–3 fold) increase risk for breast cancer [3]. As higher risks were increasingly suggested, at least for specific mutations [33], [34], the inclusion of this gene in hereditary breast cancer tests would be justified.

In our study both breast tumors from our *PALB2* families were IDC, even though one of them had a triple negative phenotype (c.1653T>A, p.Tyr551Stop), while the other was ER+ and PR+ (c.3362del, p.Gly1121ValfsX3). It has been shown that some PALB2-associated breast cancers display a more aggressive tumor phenotype, including triple-negative disease, higher tumor grade and higher Ki67 expression [35]. Thus, tumors of the 1592delT *PALB2* mutation carriers presented triple negative phenotype more often (54.5%, P<0.0001) than those of other familial (12.2%) or sporadic (9.4%) breast cancer patients [35]. In fact, nearly 40% of the PALB2-associated breast tumors identified to date displaied a triple-negative phenotype, regardless of the specific *PALB2* mutation [16]. This representation of triple negative tumors, more akin to *BRCA1*- than *BRCA2*-related tumors, could be related to the nature of the interaction and/or certain functional similarities between PALB2 and BRCA1 [11], [12], or a direct transcriptional activation of the estrogen receptor by PALB2 as has been shown for BRCA1 [36]. However, larger numbers of *PALB2*-related tumors will need to be studied before any firm conclusions can be drawn.

As shown in Table 3, different prediction tools gave contradictory results. For instance, missense variants c.656 A>G (p.Asp219Gly) and c.2590 C>T (p.Pro864Ser) were predicted as C65 (likely to be pathogenic) by A-GVGD and as Benign and Tolerated by Polyphen and Sift. Prediction of individual variants can be sensitive to the aligment used; both the number and type of orthologues aligned at the mutation site can affect prediction of pathogenicity [37]. Since we used default alignments for each tool, we cannot rule out that the different outcomes we have for these variants is related to this. However, an optimum alignment is difficult to identify and is likely to vary depending on the variant tested [37]. It has been previously shown that the accuracy of the result improves when multiple algorithms give the same prediction [38]. Considering this as well as such consensus predictions for all tools, four of the missense variants identified in our patients were classified as probably deleterious (Table 3). Three of them (c.1010C>T, c.2816T>G and c.2993G>A), had not been previously related with breast cancer risk [3], [19], [21], [24]. Although a recent study did not showed evidence of the influence of rare *PALB2* missense mutations and breast cancer risk [34], we think that multifactorial analysis incorporating different approaches to obtain a final pathogenic probability, are required to assess the implication of each of these missense *PALB2* variants.

Results from the in silico splicing analysis show three variants predicted to modify the scores for the canonical AG/GT dinucleotides, and three other variants that would modify the score of cryptic splice sites. Recently published guidelines for splicing analysis [39] indicate that only variants located in the consensus sites described by Cartegni et al [40], 11 bases for the 5′ site (from the 3 last exonic to the 8 first intronic bases) and 14 bases for the 3′ site (from the 12 last intronic to the first 2 exonic bases), would have reliable predictions with these bioinformatic tools [39]. In our study, this would mean that the unique reliable prediction is for variant c.110 G>A (p.Arg37His), at the second exonic base from the 3′ site. However, the reduction in the score predicted by the algorithms, all lower than 10%, and the absence of variations near cryptic splice sites would suggest that c.110 G>A (p.Arg37His) is not a variant producing a major impact in splicing process. The RNA analysis of the variant confirmed this prediction (Table 3).

## Conclusions

In summary, we found that *PALB2* mutations occur with a prevalence of 1.5% in a population of *BRCA1/2*-negative breast cancer patients specifically selected from a personal and/or familiar history of pancreatic cancer. This is not much different from the prevalence described for families not selected for the presence of pancreatic cancer. However, we cannot rule out a higher *PALB2* prevalence in series with more pancreatic cancer index cases.

*PALB2* mutations seem to explain only a small fraction of the clustering of both pancreatic and breast cancer. It is therefore, crucial that future research aims to identify other gene(s) that are involved in the development of familiar breast/pancreatic cancer cases.

## Supporting Information

Table S1 **Participating centers and families from Spain.**
(DOCX)

Text S1 **This document summarizes the meanings of scores of bioinformatic programs used.**
(DOCX)
