# Supplementary material for: Analysis of PALB2 Gene in BRCA1/BRCA2 Negative Spanish Hereditary Breast/Ovarian Cancer Families with Pancreatic Cancer Cases
Source: PLoS One. 2013 Jul 23;8(7):e67538. doi: 10.1371/journal.pone.0067538 (PMC3720732; doi:10.1371/journal.pone.0067538)
Supplement: Table S1 — Participating centers and families from Spain. (DOCX) [file pone.0067538.s001.docx]

**Table S1.** Participating centres and families from Spain.

| **Center/Hospital** | **Number of families** |
| --- | --- |
| Hospital Universitario San Carlos (Madrid) | 28 |
| CNIO (Madrid) | 24 |
| Hospital Universitari Vall d’Hebrón (Barcelona) | 22 |
| Hospital Infantil Miguel Servet (Zaragoza) | 13 |
| Instituto de Biología y Genética Molecular (Uva-CSIC, Valladolid) | 12 |
| Hospital de Cruces (Barakaldo) | 9 |
| Fundación Pública Galega de Medicina Xenómica (Galicia) | 9 |
| Hospital de la Santa Creu i Sant Pau (Barcelona) | 5 |
| Hospital Universitari de Sant Joan (Reus) | 4 |
| Corporació Parc Taulí-Consorci Sanitari de Terrassa (Barcelona) | 4 |
| Institut Catala d’Oncologia (Barcelona) | 2 |
| **Total** | **132** |
