# Supplementary material for: Analysis of PALB2 Gene in BRCA1/BRCA2 Negative Spanish Hereditary Breast/Ovarian Cancer Families with Pancreatic Cancer Cases
Source: PLoS One. 2013 Jul 23;8(7):e67538. doi: 10.1371/journal.pone.0067538 (PMC3720732; doi:10.1371/journal.pone.0067538)
Supplement: Text S1 — This document summarizes the meanings of scores of bioinformatic programs used. (DOCX) [file pone.0067538.s002.docx]

**Text S1.**

**This document summarizes the meanings of scores of bioinformatic programs used.**

**Splicing predictions**

SSF, MaxEnt, NNSPLICE and HSF are bioinformatic tools that use current knowledge on base composition of the splice site sequences but run distinct algorithms that generate a score matrix for each splice site. The availability of statistics for a large number of splice sites makes it possible to rate each location in a gene for its potential as a splice site. SSF (thresholds 0-100) was developed to predict potential exons in a sequence, using a scoring and a ranking scheme based on nucleotide weight tables. The MaxEnt framework (thresholds 0-12) is based on the Maximum Entropy principle and uses large datasets of human splice sites and takes into account adjacent and non-adjacent dependencies. These splice site models assign a log-odd ratio (MaxEnt score) to a 9 bp (5’ splice site) or a 23 bp (3’ splice site) sequence. The higher the score, the higher the probability that the sequence is a true splice site. NNSPLICE (thresholds 0-1) uses a computer-learning method based on neural networks that identifies sequences patterns once it is trained with a set of real splicing signals. The larger the set of real splicing signal used, the better the predictions obtained. The Human Splicing Finder (HSF) (thresholds 0-100) calculates the consensus values of potential splice sites and search for branch points. A higher consensus value represents higher strength and more possibility for the splice site to be authentic [40].

**AA substitution scoring matrices**

Polyphen-2 uses multiple sequence alignments and adds structure-based criteria to predict non-synonymous SNP phenotype with an estimated accuracy of about 80%. It is more limited when structural data are not available. Polyphen relies on annotation in the SWALL database (<http://srs.ebi.ac.uk>) to determine if an AA position is involved in metal-binding, formation of disulfide bonds, or active site catalysis. PolyPhen identifies and aligns homologs of the input sequences via BLAST search. The alignment is used to calculate a matrix of “profile scores”, which are logarithmic ratios of the likehood of this AA occurring at any position (background frequency). Profile scores of allelic variants are compared to assess whether the substitution is rarely or never observed in the protein family. The AA variant is mapped to the known 3D structure to assess whether it is likely to affect the hydrophobic core of a protein, electrostatic interactions, interactions with ligands, or other important features of a protein. If the structure of a query protein is unknown, Polyphen will use homologous proteins with known structure. PolyPhen scores were classified as “probably benign” to protein function (0.000–0.999), “borderline” to protein function (1.000–1.249), “potentially damaging” to protein function (1.250–1.499), “possibly damaging” to protein function (1.500–1.999), or “damaging” to protein function (≥2.000).

SIFT is a web-based program based on the BLOSUM62 matrix. SIFT is a multistep procedure that: 1) searches for similar sequences; 2) chooses closely related sequences that may share similar function to the query sequence; 3) aligns these chosen sequences; and 4) calculates normalized probabilities for all possible substitutions from the alignment. The normalized probability calculated to each position produce the SIFT scores, that were designated as “tolerant” (0.201–1.00), “borderline” (0.101–0.20), “potentially intolerant” (0.051–0.10), or “intolerant” (0.00–0.05).

Align-GVGD is an automated program that predicts variants in the query sequence based on a combination of Grantham variation, based on chemical differences of an observed variant relative to the evolutionary difference (Grantham deviation). The Grantham method considers composition, polarity and molecular volume. The AGVGD output classifies amino acid changes from C0 (change unlikely to be pathogenic), C15, C25, C35, C45, C55 to C65 (change most likely to be pathogenic) according to AGVGD graded classifiers used with the software.
